# Increased tibial tubercle-trochlear groove and patellar height indicate a higher risk of recurrent patellar dislocation following medial reefing

**DOI:** 10.1007/s00167-021-06581-0

**Published:** 2021-05-25

**Authors:** Marc-Daniel Ahrend, Tobias Eisenmann, Moritz Herbst, Boyko Gueorguiev, Gabriel Keller, Florian Schmidutz, Stefan Döbele, Steffen Schröter, Christoph Ihle

**Affiliations:** 1 Department of Traumatology and Reconstructive Surgery, BG Trauma Center Tübingen, Eberhard Karls University Tübingen, Tübingen, Germany; 2 AO Research Institute Davos, Davos, Switzerland; 3 Department of Diagnostic and Interventional Radiology, University Hospital Tuebingen, Tübingen, Germany; 4 Department of Traumatology and Reconstructive Surgery, Diakonie Klinikum GmbH Jung-Stilling-Krankenhaus, Siegen, Germany

**Keywords:** Patellar dislocation, Medial reefing, Recurrent dislocation, MRI

## Abstract

**Purpose:**

Identifying anatomical risk factors on recurrent dislocation after medial reefing is important for deciding surgical treatment. The present study aimed to retrospectively analyze the preoperative magnetic resonance imaging (MRI)-based parameters of patients treated with medial reefing and whether these parameters lead to a higher risk of recurrent dislocation.

**Methods:**

Fifty-five patients (18.6 ± 6.6 years) who underwent medial reefing after primary traumatic patellar dislocation (84% with medial patellofemoral ligament [MPFL] rupture) were included. Patients were followed up for at least 24 months postoperatively (3.8 ± 1.2 years) to assess the incidence of recurrent patellar dislocation. In patients without recurrent dislocation, the Kujala and subjective IKDC scores were assessed. Moreover, the tibial tubercle-trochlear groove (TT-TG), sulcus angle, patellar tilt, patellar shift, and lateral trochlea index (LTI) were measured. The patellar height was measured using the Caton-Dechamps (CDI), Blackburne-Peel (BPI), and Insall-Salvati index (ISI). The cohort was subclassified into two groups with and without recurrent dislocation. Differences between groups were analyzed with respect to the MRI parameters.

**Results:**

Forty percent had a pathological sulcus angle of > 145°, 7.2% had an LTI of < 11°, 47.3% had a patellar tilt of > 20°, and 36.4% had a TT-TG of ≥ 16 mm. Increased patellar height was observed in 34.5, 65.5, and 34.5% of the patients as per CDI, BPI, and ISI, respectively. Nineteen (34.5%) patients suffered from recurrent dislocation. Compared with patients without recurrent dislocation, those with recurrent dislocation had a significantly lower LTI (*p* = 0.0467). All other parameters were not significantly different between the groups. Risk factor analysis showed higher odds ratios (OR > 2), although not statistically significant, for MPFL rupture (OR 2.05 [95% confidence interval 0.38–11.03], LTI (6.6 [0.6–68.1]), TT-TG (2.9 [0.9–9.2]), and patellar height according to ISI (2.3 [0.7–7.5]) and CDI (2.3 [0.7–7.5])). Patients without recurrent dislocation had a Kujala score of 93.7 ± 12.1 (42–100) points and an IKDC score of 90.6 ± 11.7 (55.2–100) points.

**Conclusion:**

Anatomical, MRI-based parameters should be considered before indicating medial reefing. A ruptured MPFL, an LTI < 11°, a TT-TG ≥ 16 mm, a patellar tilt > 20 mm, and an increased patellar height according to ISI and CDI were found to be associated, although not significantly, with a higher risk (OR > 2) of recurrent patellar dislocation after medial reefing. Thorough preoperative analysis is crucial to reduce the risk of recurrent dislocation in young patient cohorts.

**Level of evidence:**

Level IV

## Introduction

Patellar dislocation is a common knee injury with a primary patellar dislocation incidence of up to 108/100,000 in Western Europe between the age of 10 and 19 years [19, 45]. The first dislocation is often a result of a traumatic event and can lead to recurrent patellar dislocation with a reduced quality of life [27]. Recurrent patellar dislocation is reported in 22.7–29.4% depending on the injury pattern, various anatomical risk factors assessed by magnetic resonance imaging (MRI), or radiographs as well as the applied treatment option [15, 18, 19].

Thus, there is no single gold standard or treatment algorithm available for treating primary patellar dislocation. Whether it can be treated surgically or non-surgically is still a subject of debate, and there is insufficient evidence to support either of the options [40, 44]. According to the best available evidence, surgical treatment of acute patellar dislocation may result in a lower rate of recurrent dislocations than non-surgical treatment; however, functional outcome scores are not improved by surgical treatment [15, 35]. Surgical reconstruction of the medial patellofemoral ligament (MPFL) is widely performed, and there are several publications available that report different techniques, outcomes after treatment, and the analysis of the potential risk factors of recurrent dislocation [6, 16, 30, 31, 41]. By contrast, medial reefing is a less invasive procedure but also less evaluated. Moreover, good clinical outcome has been reported following this treatment [20]. Nevertheless, the indications and contraindications of medial reefing have been rarely described, and individual anatomy that predisposes one to primary or recurrent dislocation has not been comprehensively considered. In particular, anatomical parameters based on MRI scans before medial reefing have not been described yet. Moreover, an analysis of potential anatomical risk factors on recurrent dislocation after medial reefing is still lacking.

Therefore, this study primarily aimed to retrospectively analyze the preoperative MRI-based parameters of patients who were treated with medial reefing to provide further information on the indication of medial reefing. The secondary aim of this study was to analyze whether these MRI-based anatomical parameters influence the risk of recurrent dislocation after medial reefing. It was hypothesized that patients with trochlear dysplasia and increased patellar height were more likely to experience recurrent dislocation after medial reefing.

## Materials and methods

**Fig. 1:**
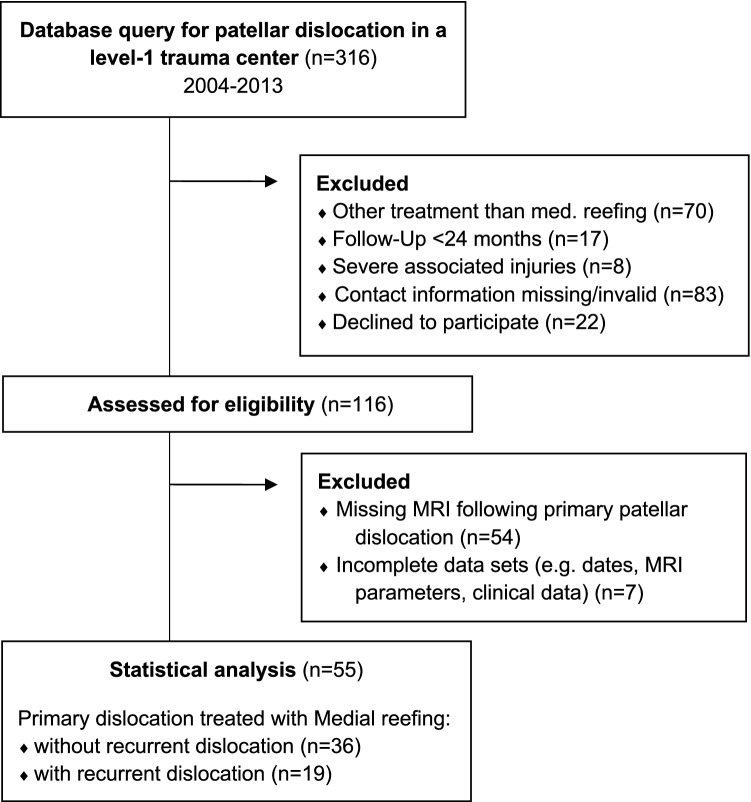
Patient flow chart with exclusion criteria

The local ethics committee (195/2014BO2) reviewed and approved the study protocol, and informed consent was obtained from all included subjects. Between 2004 and 2013, 316 patients presented with primary patellar dislocation at a level-1 trauma center in Western Europe. Patients with primary patellar dislocation and initial medial reefing were included, whereas patients with missing data, including a missing MRI scan directly following primary patellar dislocation, or other treatments, such as non-surgical treatment, MPFL reconstruction, or an osteotomy, were excluded. Patients were followed up for at least 24 months postoperatively (3.8 ± 1.2 [range 2.1–7.5 years]) to assess the incidence of recurrent patellar dislocation following medial reefing. During the follow-up, the Kujala [13] and the subjective IKDC scores [23] were assessed in patients without recurrent dislocation to analyze functional outcome after medial reefing. The patient flow chart is presented in Fig. 1.

### Arthroscopic medial reefing

The indication for the surgical treatment of patellar dislocation was a traumatic patellar dislocation. Surgery was performed in an average of 38.7 ± 50.3 days following the primary dislocation. Surgery was performed as described by Ihle et al. [20]. A Vicryl CTX sized 0 suture (Johnson & Johnson Medical GmbH, Ethicon Germany, Norderstedt, Germany) was placed at the proximal and medial borders of the patella directly next to the bone and taken out approximately 3 cm posteromedial. A small skin incision at the insertion of the suture was performed, and the suture was passed percutaneously via the capsule to the extra-articular area. Then, with the same technique, a second and a third suture were positioned 1.5 cm distally to the above suture. The knot was tightened after removing the intra-articular fluid at 30° knee flexion.

Full weight-bearing was allowed immediately after surgery. The active and passive range of motion was limited between 0° and 60° for the first 3 weeks and 0° and 90° for additional 3 weeks; return-to-sport was allowed after 12 weeks.

### MRI evaluation

**Fig. 2:**
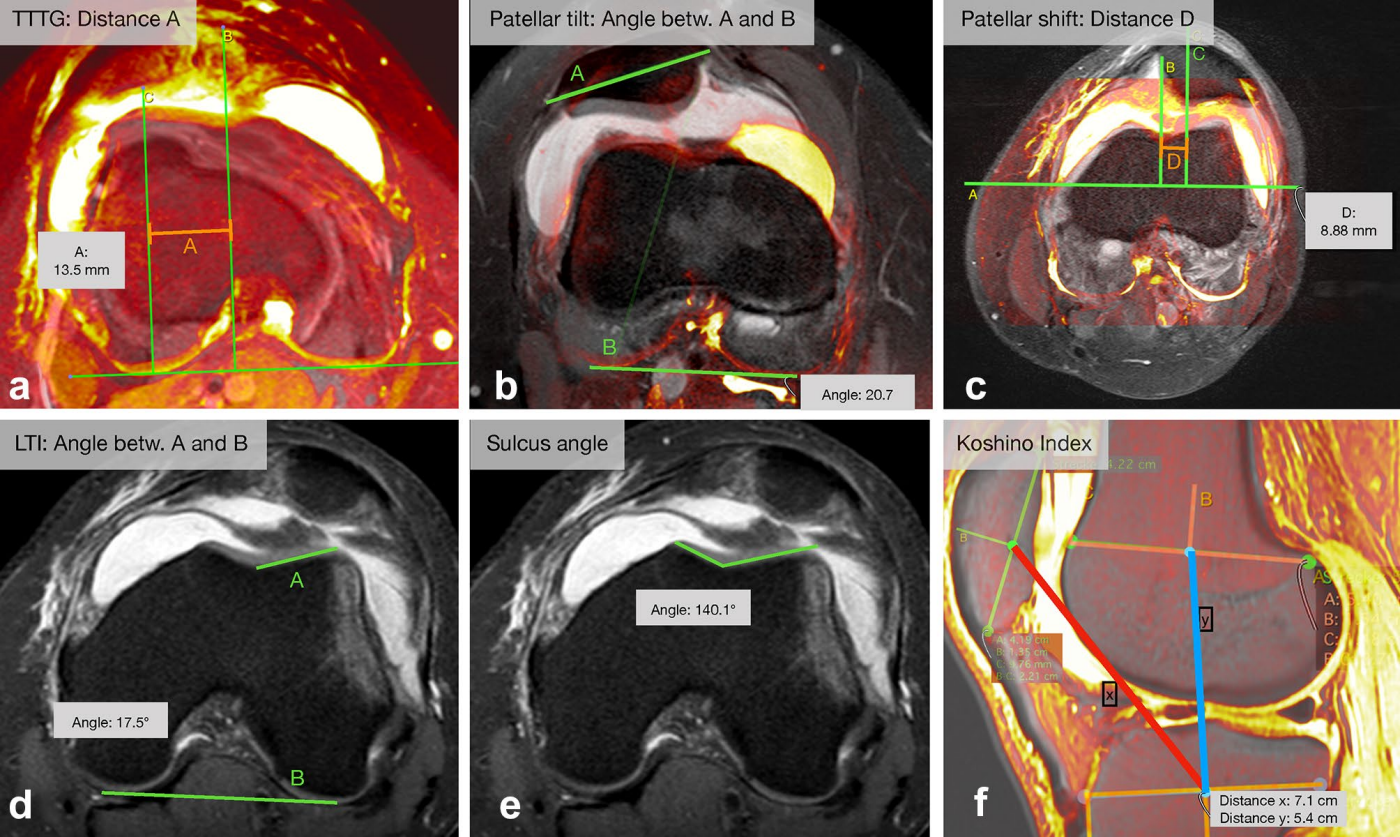
Overview of MRI parameters measured with Osirix. **a** Overlapped axial slices to measure TT-TG (tibial tubercle-trochlear groove), **b** patellar tilt **c** patellar shift. **d** axial images to measure LTI (lateral trochlear index), **e** the sulcus angle. **f** sagittal image to measure the Koshino index

All MRI scans were analyzed by a radiologist with respect to bone bruises, cartilage lesions, and ruptures of MPFL or the medial retinaculum. The following MRI-based radiological parameters were selected based on commonly cited literature [2, 37] and measured twice by two orthopedic surgeons independently at two different time points (2 weeks interval): tibial tubercle-trochlear groove (TT-TG) [38], sulcus angle [36], patellar tilt [14], patellar shift [37], lateral trochlea index (LTI) [10] (Fig. 2), and patellar height parameters (patellotrochlear index [PTI) according to Biedert and Albrecht [4], Koshino index, Caton–Dechamps index [CDI], Blackburne–Peel index [BPI], Insall–Salvati index [ISI]). The imaging software Osirix (Pixmeo, Geneva, Switzerland) was used for analysis. The observers were blinded to the incidence of recurrent dislocation. The measurement accuracy for distances (mm) and angles was one decimal. Patellar height indices were expressed with two decimals.

TT-TG was measured on two axial slices according to Schoettle et al. [38] between the deepest cartilaginous point of the trochlear groove and the tibial tuberosity at the patellar insertion. A baseline was drawn tangent to the cartilaginous border of the posterior condyles and a perpendicular line (B) to the deepest cartilaginous point of the trochlear groove. A second image was superimposed over the first image. The second image showed the attachment of the patellar tendon. A line (C) was drawn parallel to the first line via the center of the patellar insertion. Then, the distance (A) between the perpendicular lines B and C was measured (TT-TG).

Patellar tilt was measured on an axial view as per Dejour et al. [14]. A baseline (B) was drawn tangent to the cartilaginous border of the posterior condyles. On a second image depicting the greatest patellar width, a line (A) was drawn from the medial to the lateral border of the patella. The angle between both lines was defined as the patellar tilt.

To measure patellar shift, the distance between the two parallel lines was measured. Line B was drawn via the deepest point of the trochlear groove perpendicular (A) to the posterior femoral condyle aspect. Line C was parallel to B and via the most posterior aspect of the retropatellar surface [37].

According to Carrillon et al. [10], LTI describes the morphology of the trochlear groove. It was determined by the angle between the posterior aspect of the femoral condyles (line B) and the cartilaginous surface of the lateral trochlea (A). Line A was drawn on the first craniocaudal axial view showing full articular cartilage over the lateral trochlear facet.

The sulcus angle of the trochlear groove was determined using the first transverse craniocaudal image that shows a complete cartilaginous trochlear surface. It describes the angle between the medial and the lateral aspect of the trochlear groove [33, 36].

Besides CDI [11], BPI [7], and ISI [22], PTI as per Biedert and Albrecht [4] was measured in sagittal slices. PTI is the overlap percentage of the patellar cartilage (line A) and the trochlear cartilage (E). The trochlear cartilage is measured parallel to the patellar cartilage from the superior aspect of the trochlear cartilage to a reference line perpendicular to the inferior end of line A. The Koshino index was also measured in sagittal slices. Three lines were drawn, and the middle of each line was determined as follows: a line drawn from the tip to the bottom of the patellar surface, a line drawn from the anterior to the posterior cortex of the epiphysis of the tibia, and a line drawn from the posterior to the anterior cortex of the epiphysis of the femur. The Koshino index was defined as the ratio between the middle of the patella line to the middle of the tibia line (X) and the middle of the tibia line to the middle of the femoral line (Y) [28].

### Statistical analysis

The intra-observer reliability was determined between the first and second observation of Observer 1, and the interobserver reliability was determined between Observation 1 of both observers by calculating the intra-class correlation coefficient (ICC) of all MRI parameters. ICC and 95% confidence intervals (CI) were based on a two-way mixed-effects model (ICC [1, 3]). ICC values were interpreted as suggested by Shrout and Fleiss [39]. An ICC of 0.50–0.75 indicated moderate reliability; ICC values of > 0.75 indicated excellent reliability.

The mean of the four measurements (first and second observations of observers 1 and 2) was measured for each MRI-based parameter and used for further calculations. The means of these parameters as well as demographic data were descriptively analyzed. Data are presented as absolute and relative values (*n* (%)) as well as means ± standard deviation (minimum–maximum). The cohort was subclassified into two groups: patients with and without recurrent dislocation. Data distribution was analyzed using the Shapiro–Wilk test, and groups were tested with respect to differences in demographic and MRI parameters. Independent *t* tests were used for normally distributed data. The Mann–Whitney *U* test was used when data were not normally distributed. Bivariate data were tested using the Chi-square test. For each MRI parameter, a threshold between the normal and abnormal value was defined based on literature recommendation (Table 1). The odds ratio (OR) of each factor was calculated using contingency tables.

**Table 1:** Distribution of preoperative MRI parameters in patients underwent medial reefing (mean ± SD (minimum–maximum) or *n* (%))

	Reference/normal values	Number (%) of patients with abnormal values	Mean ± SD (min.–max.)
Sulcus Angle (°)	≤ 145° [47]	22 (40.0%)	142.7 ± 6.0 (126.8–152.6)
Lateral trochlea index (°)	> 11° [10, 25]	4 (7.2%)	17.6 ± 5.0 (7.1–32.4)
TT-TG (mm)	< 16 mm [3]	20 (36.4%)	14.4 ± 4.1 (4.9–22.6)
Patella tilt (°)	≤ 20° [3, 14]	26 (47.3%)	20.2 ± 7.0 (7.8–41.7)
Patella shift (mm)	< 2.5 mm [37]	47 (85.5%)	7.2 ± 4.4 (−0.6–21.0)
Patellar height			
Patellotrochlea index	0.13–0.50 [4]	0	0.47 ± 0.16 (0.19–0.89)
Koshino index	0.99–1.20 [28]	38 (69.1%)	1.25 ± 0.07 (1.04–1.39)
CDI	Patella alta: > 1.3 [47]	39 (34.5%)	1.23 ± 0.13 (0.97–1.51)
BPI	Patella alta: > 1.0 [5]	36 (65.5%)	1.06 ± 0.15 (0.80–1.34)
ISI	Patella alta: > 1.3 [1]	19 (34.5%)	1.20 ± 0.18 (0.78–1.56)

The significance level was set at 5%. Statistical analysis was performed using JMP® (SAS Institute Inc., JMP®, Version 13.0.0, Cary, NC, USA) and STATA® (Stata Corporation, 15.0, College Station, TX, USA).

## Results

Fifty-five patients (18.6 ± 6.6 years [range 10–43], female: 23 [41.8%], male: 32 [58.2%]) were included in the study, and they underwent medial reefing after primary patellar dislocation.

MRI parameters are presented in Table 1; they showed moderate to excellent intra- and interobserver reliability (Table 2). Forty-four (80.0%) patients had bone bruises at the femoral condyle, whereas 42 (76.4) had bone bruises at the patella A cartilage lesion at the patellar was identified in 31 patients (56.4%) and at the femoral condyle in 22 (40.0%) patients. MRI revealed an osteochondral flake in 14 (25.5%) patients, an MPFL rupture in 46 (83.6%), and a medial retinaculum rupture in 43 (78.2%).

**Table 2:** Intra- and interobserver reliability (ICC (95%CI)) and intra- and interobserver difference (mean ± standard deviation (minimum–maximum))

	Intraobserver reliability	Intraobserver differences − absolute values	Interobserver reliability	Interobserver differences − absolute values
Sulcus Angle	0.88 (0.80–0.93)	2.7 ± 2.9 (0–14.0)	0.74 (0.69–0.84)	3.8 ± 2.7 (0.1–11.1)
LTI	0.84 (0.74–0.90)	2.2 ± 2.0 (0–10.3)	0.86 (0.78–0.92)	2.3 ± 1.6 (0.4–7.6)
TT-TG	0.90 (0.83–0.94)	1.7 ± 1.2 (0.2–5.2)	0.82 (0.71–0.89)	2.0 ± 1.7 (0–9.2)
Patella tilt	0.93 (0.88–0.96)	2.7 ± 2.2 (0.2–12.7)	0.92 (0.87–0.95)	2.3 ± 2.1 (0.1–10.4)
Patella shift	0.94 (0.89–0.96)	1.3 ± 1.1 (0.1–5.1)	0.89 (0.82–0.94)	1.7 ± 1.3 (0–7.1)
PTI	0.81 (0.70–0.89)	0.05 ± 0.08 (0–0.56)	0.81 (0.69–0.88)	0.06 ± 0.09 (0–0.64)
Koshino index	0.79 (0.70–0.87)	0.04 ± 0.04 (0–0.22)	0.52 (0.29–0.69)	0.04 ± 0.08 (0–0.57)
CDI	0.72 (0.56–0.83)	0.08 ± 0.07 (0–0.39)	0.85 (0.76–0.91)	0.07 ± 0.05 (0–0.23)
BPI	0.78 (0.64–0.86)	0.08 ± 0.09 (0–0.39)	0.76 (0.62–0.85)	0.08 ± 0.08 (0–0.42)
ISI	0.89 (0.81–0.93)	0.06 ± 0.10 (0–0.34)	0.94 (0.90–0.96)	0.05 ± 0.04 (0–0.20)

In the cohort, 40.0% of the patients had a pathological sulcus angle of > 145°, 7.2% had a low LTI of < 11°, 47.3% had a patellar tilt of > 20°, and 36.4% had a TT-TG of ≥ 16 mm. Increased patellar height was identified in 34.5% of the patients according to CDI, 65.5% as per BPI, 34.5% according to ISI, 69.1% as per the Koshino index, and no patient had an increased patellar height according to PTI.

Nineteen (34.5%) patients who underwent medial reefing experienced recurrent patellar dislocation. Patients with recurrent dislocation had a significantly lower LTI than those without recurrent dislocation (*p* = 0.0467). All other parameters did not differ significantly between patients with and without recurrent patellar dislocation (Table 3). Risk factor analysis (Table 4) revealed no significant risk factor in the cohort, which was expressed by the CI including 1. Parameters with the largest ORs included LTI, TT-TG, and patellar height as per ISI and CDI. Moreover, patients with ruptured MPFL (OR 2.05 [95% CI 0.38–11.03]) or ruptured medial reticulum (OR 1.78 [95% CI 0.42–7.54]) were more likely to suffer from a recurrent dislocation. Patients without recurrent dislocation had a Kujala score of 93.7 ± 12.1 (42–100) and a subjective IKDC score of 90.6 ± 11.7 (55.2–100) points.

**Table 3:** Distribution of MRI parameters in patients with and without recurrent dislocation (mean ± SD (95% CI); *n* (%))

	No recurrent dislocation	Recurrent dislocation	*p* value
Age (years)	18.7 ± 6.4 (16.5–20.8)	18.5 ± 7.2 (15.0–22.0)	n.s
Female	Male: 22 (66.1%) Female: 14 (38.9%)	Male: 10 (52.6%) Female: 9 (47.4%)	n.s
Rupture of the MPFL	29 (80.6%)	17 (89.5%)	n.s
Postoperative follow-up (years)	3.9 ± 1.0 (3.5–4.2)	3.7 ± 1.5 (2.8–4.7)	
Sulcus Angle (°)	144.1 ± 5.8 (139.9–144.0)	142.0 ± 6.1 (141.3–146.9)	n.s
Lateral trochlea index (°)	18.6 ± 4.9 (16.9–20.2)	15.8 ± 4.7 (13.5–18.1)	0.0467
TT-TG (mm)	13.8 ± 3.5 (12.6–15.0)	15.6 ± 4.8 (13.3–18.0)	n.s
Patella tilt (°)	18.9 ± 5.6 (17.0–20.8)	22.6 ± 8.6 (18.5–26.8)	n.s
Patella shift (mm)	6.8 ± 3.5 (5.6–8.0)	8.0 ± 5.7 (5.2–10.7)	n.s
Patellar height			
Patellotrochlea index (%)	0.49 ± 0.17 (0.43–0.54)	0.44 ± 0.14 (0.37–0.51)	n.s
Koshino index	1.24 ± 0.08 (1.21–1.26)	1.26 ± 0.07 (1.23–1.30)	n.s
CDI	1.22 ± 0.12 (1.18–1.26)	1.25 ± 0.16 (1.18 -1.33)	n.s
BPI	1.05 ± 0.13 (1.00–1.09)	1.08 ± 0.17 (1.00–1.16)	n.s
ISI	1.19 ± 0.17 (1.13–1.25)	1.23 ± 0.18 (1.14–1.32)	n.s

**Table 4:** Distribution of risk factors in the MRI in terms of defined thresholds of pathologic values

	Normal vs. abnormal	No recurrent dislocation	Recurrent dislocation	OR (95% CI)
Age	≥ 18 < 18	13 (63.9%) 23 (36.1%)	8 (42.1%) 11 (57.89%)	1.29 (0.41–4.01)
Gender	Male Female	22 (61.1%) 14 (38.9%)	10 (52.6%) 9 (47.4%)	1.41 (0.46–4.35)
Sulcus Angle (°)	≤ 145 > 145°	23 (63.9%) 13 (36.1%)	10 (52.6%) 9 (47.4%)	1.59 (0.52–4.92)
Lateral trochlea index (°)	≥ 11° < 11°	35 (97.2%) 1 (2.8%)	16 (84.2%) 3 (15.8%)	6.56 (0.63–68.07)
TT-TG (mm)	< 16 mm ≥ 16 mm	26 (72.2%) 10 (27.8%)	9 (47.4%) 10 (52.6%)	2.89 (0.91–9.20)
Patella tilt (°)	≤ 20° > 20°	21 (58.3%) 15 (41.7%)	8 (42.1%) 11 (57.9%)	1.93 (0.62–5.94)
Patella shift (mm)	≤ 2.5 mm > 2.5 mm	5 (13.9%) 31 (86.1%)	3 (15.8%) 13 (84.2%)	0.86 (0.18–4.10)
Patellar height				
Patellotrochlea index	≥ 0.13 < 0.13	36 (100%) 0	19 (100%) 0	*
Koshino index	≤ 1.2 > 1.2	12 (33.3%) 24 (66.7%)	5 (26.3%) 14 (73.7%)	1.4 (0.41–4.81)
CDI	≤ 1.3 > 1.3	26 (72.2%) 10 (27.8%)	10 (52.6%) 9 (47.4%)	2.34 (0.73–7.46)
BPI	≤ 1.0 > 1.0	13 (36.1%) 23 (63.9%)	6 (31.6%) 13 (68.4%)	1.22 (0.38–4.00)
ISI	≤ 1.3 > 1.3	26 (72.2%) 10 (27.8%)	10 (52.6%) 9 (47.4%)	2.34 (0.73–7.46)

(*OR could not be calculated)

## Discussion

The most important finding of the present study was that a ruptured MPFL, an LTI of < 11°, a TT-TG of ≥ 16 mm, and an increased patellar height as per ISI and CDI results in a higher risk (OR > 2) of recurrent patellar dislocation after medial reefing, although the results were not statistically significant. Furthermore, a moderate proportion of patients showed pathological and radiological parameters associated with trochlea dysplasia (sulcus angle > 145°: 40%; LTI < 11°: 7.2%) and increased patellar height (CDI > 1.3: 35%; ISI > 1.3: 35%).

Medial reefing has been less evaluated than the MPFL reconstruction or non-surgical treatment of patellar dislocation. In particular, there is no detailed analysis of preoperative MRI parameters that describe indications for medial reefing as well as that evaluate predisposing anatomical factors of recurrent patellar dislocation. Literature describes surgical indications for this procedure mostly based on radiographs. Normal Q-angles and tubercle–sulcus angles as well as no excessive patellar tilt during examination are requirements for medial reefing. A contraindication for medial reefing is a lateral patellofemoral angle on a Merchant radiograph that opens rather medially than laterally [32]. Bodulla et al. [8] described normal bony alignment in addition to the supine *Q*-angles of < 20°, seated tubercle angles of ≤ 5° and ISI of < 1.2. Cerciello et al. [12] provided a more comprehensive preoperative assessment of radiological parameters before medial reefing. Imaging, including the weight-bearing anterior–posterior and lateral radiographs of the knee joint, bilateral skyline views at 30° of flexion, and computed tomography, was performed to assess TT-TG distance and the patellar tilt. MRI is frequently used following patellar dislocation and is important to evaluate anatomical risk factors and concomitant injuries, such as cartilage lesions or osteochondral flakes [43]. This is of particular importance in patients treated with medial reefing because the surgical procedure does not modify these anatomical factors.

Trochlear dysplasia, patellar height, and patellar tilt are known risk factors for primary dislocation. Ridley et al. [34] summarized studies that analyzed anatomical patellofemoral instability imaging measurements in patients with patellofemoral instability and healthy controls. The healthy control group and patients with patellofemoral instability differed with respect to ISI (1.10 [95% CI 1.06–1.15] vs. 1.25 [1.22–1.29]), CDI (1.03 [0.93–1.13] vs. 1.24 [1.17–1.31]), patellar tilt (9.19° [6.58°–11.8°] vs. 19.7° [11.3°–28.2°]), TT-TG (9.23 [8.22–10.2 mm] vs. 13.9 [11.6–16.1 mm]), and the sulcus angle (149° [136°–162°) vs. 157° (152°–162°]). These patients with a patellar dislocation or patellar instability showed similar values compared with those in the present cohort.

Several authors [2, 16, 27] described factors associated with a higher risk of recurrent dislocation for different primary treatments, e.g., MPFL reconstructions are more likely to fail in patients with patella alta and an increased TT-TG distance [2, 14, 27]. Arendt et al. [1] analyzed 145 patients and observed that skeletal immaturity, high sulcus angle, and large ISI were significant predictors of recurrent dislocation in patients who underwent non-surgical treatment. Moreover, the cutoff points of a sulcus angle of ≥ 154° and ISI of ≥ 1.3 were found. In the presence of these factors, there was a probability of 23% of recurrent dislocation. Zhang et al. [49] analyzed 166 patients and observed that trochlear dysplasia, elevated TT-TG distance, and patella alta were independently associated with a higher incidence of a second patellar dislocation.

The anatomical risk factors have not been addressed for both non-surgical treatment and medial reefing after primary dislocation. Thus far, there has been no analysis of MRI parameters of patients with medial reefing. Despite the relatively small sample size, similar risk factors for recurrent dislocation compared with non-surgical treatment were found in this study. Similar to Balcarek et al. [3], ORs were calculated to evaluate the influence of MRI parameters on the risk of recurrent dislocation. Balcarek et al. [3] interpreted an OR of > 1.3 as having a relevant effect on recurrent dislocation, although their results and the results of the present study were not statistically significant. The interpretation of these variables within a surgical algorithm has to be defined [2] and other factors, including activity level, torsional deformities, age, and gender, also need to be considered [26, 29].

The study has several limitations. First, the present study did not include any control or comparison group. It is unclear whether medial reefing is justified for treating primary patellar dislocation, particularly considering the low recurrent dislocation rates of MPFL reconstruction [9, 17, 24, 46]. Because of the retrospective nature of the present study, selection bias might have occurred leading to an overestimation of the recurrent dislocation rate; it was easier to include patients treated in our clinic owing to recurrent dislocation after medial reefing and to collect MRI data. Moreover, torsional and frontal alignment were not analyzed but are important factors that should be considered [48]. Patients with recurrent dislocation should receive long-leg weight-bearing, and Merchant radiographs, torsional computed tomography, and MRI should be performed to comprehensively assess the anatomical risk factors of patellar dislocation. Moreover, measurements might have been altered, such as the patellar tilt, because of swelling and effusion [3].

Cutoff values for categorizing between the normal and abnormal values were chosen based on literature analysis but could have altered statistical findings because the mean differences of MRI parameters between patients with and without recurrent dislocations were small (Table 3). It is difficult to define thresholds because patients without patellar dislocation can have abnormal values, and distinguishing risk factors requires even higher sample sizes. Even in large meta-analysis, the CIs of anatomical risk factors of patients with and without patellar instability are mostly overlapping [34]. Furthermore, differences in these cutoff values can be found frequently between studies, and no consensus has been found yet [5]. In particular, regarding the clinical impact of the cutoff values, further research is needed. In addition, cutoff values are dependent on the image modality [36, 42]. Moreover, the analysis of risk factors is complex because of the interaction of parameters [21]. No statistical corrections for multiple comparisons were calculated. A receiver operating characteristic curve to calculate cohort related cutoff values and multiple regression analysis to distinguish among the influences of different factors could not be performed owing to the relatively small sample size of the present study.

## Conclusion

Anatomical, MRI-based parameters should be considered before indicating medial reefing. A ruptured MPFL, an LTI of < 11°, a TT-TG of ≥ 16 mm, a patellar tilt of > 20 mm, and an increased patellar height according to ISI and CDI were found to be associated, although not significantly, with a higher risk (OR > 2) of recurrent patellar dislocation after medial reefing. Thorough preoperative analysis is crucial to reduce the risk of recurrent dislocation in young patient cohorts.
